# Inhibition of neutrophil elastase attenuates airway hyperresponsiveness and inflammation in a mouse model of secondary allergen challenge: neutrophil elastase inhibition attenuates allergic airway responses

**DOI:** 10.1186/1465-9921-14-8

**Published:** 2013-01-24

**Authors:** Hikari Koga, Nobuaki Miyahara, Yasuko Fuchimoto, Genyo Ikeda, Koichi Waseda, Katsuichiro Ono, Yasushi Tanimoto, Mikio Kataoka, Erwin W Gelfand, Mitsune Tanimoto, Arihiko Kanehiro

**Affiliations:** 1 Department of Hematology, Oncology, Allergy and Respiratory Medicine, Okayama University Graduate School of Medicine, Dentistry, and Pharmaceutical Sciences, Okayama, Japan; 2 Department of Pediatrics, Division of Cell Biology, National Jewish Health, Denver, Colorado, USA

**Keywords:** Neutrophil, Elastase, Airway, Hyperresponsiveness, Asthma

## Abstract

**Background:**

Chronic asthma is often associated with neutrophilic infiltration in the airways. Neutrophils contain elastase, a potent secretagogue in the airways, nonetheless the role for neutrophil elastase as well as neutrophilic inflammation in allergen-induced airway responses is not well defined. In this study, we have investigated the impact of neutrophil elastase inhibition on the development of allergic airway inflammation and airway hyperresponsiveness (AHR) in previously sensitized and challenged mice.

**Methods:**

BALB/c mice were sensitized and challenged (primary) with ovalbumin (OVA). Six weeks later, a single OVA aerosol (secondary challenge) was delivered and airway inflammation and airway responses were monitored 6 and 48 hrs later. An inhibitor of neutrophil elastase was administered prior to secondary challenge.

**Results:**

Mice developed a two-phase airway inflammatory response after secondary allergen challenge, one neutrophilic at 6 hr and the other eosinophilic, at 48 hr. PAR-2 expression in the lung tissues was enhanced following secondary challenge, and that PAR-2 intracellular expression on peribronchial lymph node (PBLN) T cells was also increased following allergen challenge of sensitized mice. Inhibition of neutrophil elastase significantly attenuated AHR, goblet cell metaplasia, and inflammatory cell accumulation in the airways following secondary OVA challenge. Levels of IL-4, IL-5 and IL-13, and eotaxin in BAL fluid 6 hr after secondary allergen challenge were significantly suppressed by the treatment. At 48 hr, treatment with the neutrophil elastase inhibitor significantly reduced the levels of IL-13 and TGF-β1 in the BAL fluid. In parallel, in vitro IL-13 production was significantly inhibited in spleen cells from sensitized mice.

**Conclusion:**

These data indicate that neutrophil elastase plays an important role in the development of allergic airway inflammation and hyperresponsiveness, and would suggest that the neutrophil elastase inhibitor reduced AHR to inhaled methacholine indicating the potential for its use as a modulator of the immune/inflammatory response in both the neutrophil- and eosinophil-dominant phases of the response to secondary allergen challenge.

## Background

Bronchial asthma is a complex syndrome characterized by airway obstruction, airway inflammation and airway hyperresponsiveness (AHR) [1]. In the pathogenesis of asthma, various inflammatory cells contribute to the development of AHR and allergic airway inflammation. A common theory is that the disease results from chronic airway inflammation leading to AHR and reversible airway obstruction [2]. In adults with stable asthma treated with inhaled corticosteroids, ~40% have eosinophilic asthma, whereas 25% have neutrophilic asthma [3], and asthmatics with neutrophilic airway inflammation also have AHR, along with many other phenotypes of asthma. Neutrophils are one of the pro-inflammatory cell types whose role in the pathology of asthma has been emphasized recently. Acute severe asthma has been shown to be associated with neutrophilic infiltration of the airways [4,5]. Neutrophils were reported to predominate in fatal attacks of short duration [3], and in the early stages of status asthmaticus, neutrophilic infiltration of the airways was demonstrated [6,7]. However, the specific role of neutrophils in the pathogenesis of asthma has not been clarified.

We have previously shown important differences when a primary challenge approach was compared with mice that had previously been sensitized and challenged and later provoked with a single airway challenge (secondary challenge) [8]. It has been shown that neutrophils increase in bronchoalveolar lavage (BAL) fluid 6 hours after provocation, whereas eosinophils increase 48 hours after provocation [9]. This model perhaps more closely mimics the human situation of previous exposure than primary models of acute exposure.

Among the neutrophil proteases, elastase has the greatest potential to cause tissue injury and alter airway function [10]. Neutrophil elastase has been shown to play an important role in neutrophil-endothelial adhesion and extravasation elicited by pro-inflammatory mediators [11]. Association of neutrophil elastase activity with asthmatic subjects has been reported [12-14]. Recent study demonstrated that significant increases of sputum IL-8 and neutrophil elastase protein and IL-8 receptor gene expression were shown in the neutrophilic asthma and systemic inflammation was increased in patients with neutrophilic airway inflammation and associated with worse clinical outcomes [15]. Goblet cell degranulation was inhibited when neutrophil recruitment was prevented or when neutrophil elastase activity was inhibited after antigen challenge of sensitized guinea pigs [16,17]. In Ascaris suum allergen-induced sheep model, Fujimoto et al. reported that the neutrophil elastase inhibitor, ONO-5046, reduced asthmatic responses but did not affect the number of eosinophils and lymphocytes in BAL fluid [18]. In this study, we evaluated the role of neutrophil elastase in allergen-induced inflammation and AHR on a background of previously established disease, provoked by secondary challenge.

In established asthma, the importance of neutrophil elastase on allergen-induced AHR and airway inflammation has not been elucidated. In addition, the mechanisms whereby neutrophil elastase affects allergic airway responses and inflammation remain to be identified. In the present study, to define the role of neutrophil elastase following established allergen-induced AHR and inflammation and response to secondary challenge, we utilized sivelestat, a specific synthetic inhibitor of neutrophil elastase.

## Material and methods

### Animals

Female BALB/c mice (8–10 wk of age) were purchased from Charles River Japan, Inc. (Yokohama, Japan). The mice were maintained on diets free of ovalbumin (OVA). All experimental animals used in this study were housed under constant temperature and light cycles and experiments performed under a protocol approved by the Institutional Animal Care and Use Committee of Okayama University Medical School (Okayama, Japan).

### Sensitization and airway challenge

Mice were sensitized by intraperitoneal injection of 20 μg of OVA (Grade V; Sigma-Aldrich, St. Louis, MO) emulsified in 2.25 mg aluminum hydroxide (AlumImuject; Pierce, Rockford, IL) in a total volume of 100 μL on days 0 and 14. Mice were challenged (primary challenge) via the airways with OVA (1% in saline) for 20 min on days 28, 29 and 30 using ultrasonic nebulizer. On day 72, mice received a single secondary challenge *via* the airways with 1% OVA for 20 min. Mice were studied 6 and 48 hr after the secondary challenge [8].

### Administration of the neutrophil elastase inhibitor

Sivelestat (N-[2-[4-(2,2-dimethylpropionyloxy)-phenylsulfonylamino]benzoyl] aminoacetic acid) is a specific synthetic inhibitor of neutrophil elastase (Ono Pharmaceutical Co., Osaka, Japan). Mice received sivelestat, 100 mg/kg/day intraperitoneally, from day 68 through to the day of study. Control groups of mice received saline in the same fashion.

### Determination of airway responsiveness

A flexiVent small-animal ventilator (SCIREQ, Montreal, PQ, Canada) was used to assess airway function (Snapshot) in anesthetized (intraperitoneal injection of sodium pentobarbital, 70 mg/kg), mechanically ventilated animals, measuring changes in lung resistance (RL) in response to increasing doses of inhaled methacholine (MCh) [18]. Airway responsiveness was assessed (150 breaths/min, tidal volume: 10 ml/kg) as a change in airway function after challenge with aerosolized MCh administered for 10 s (60 breaths/min, tidal volume: 20 ml/kg) in increasing concentrations (0, 3.125, 6.25, 12.5 and 25 mg/ml). Baseline RL values in response to saline at 6 and 48 hr were first determined. The data of RL was continuously collected for up to 3 min and maximum values were taken.

### Bronchoalveolar lavage

After assessment of AHR, lungs were lavaged via the tracheal tube with Hanks’ balanced salt solution (2 × 1 ml, 37°C). The volume of collected BAL fluid was measured in each sample and the number of BAL fluid cells was counted. Cytospin slides were stained and differentiated in a blinded fashion by counting at least 200 cells under light microscopy. BAL fluid supernatants were collected and stored at −30°C until measurement [19].

### Histological and immunohistochemistry studies

After obtaining the BAL fluid, lungs were fixed in 10% formalin. Blocks of lung tissue were cut around the main bronchus and embedded in paraffin blocks. Tissue sections 4 μm thick were affixed to microscope slides and deparaffinized. Lung sections were stained with hematoxylin-eosin (H&E) especially for analyzing the difference between eosinophils and neutrophils (final magnification:x1000), periodic acid Schiff (PAS) for identification of mucus-containing cells (goblet cells) (final magnification:x1000) and granulocyte-differentiation antigen (Gr-1) monoclonal antibody (BioLegend, San Diego, CA) [20] for detection of neutrophil infiltration (final magnification:×400) and confirmed H&E stained data. In hematoxylin-eosin and anti-Gr-1 monoclonal antibody stained lung section, the number of eosinophils, lymphocytes, and neutrophils per square millimeter in the peribronchial and perivascular tissue were analyzed using NIH Image Analysis system. More than 10 bronchioles in a minimum of 10 high-power fields per lung tissue were randomly examined in a blinded fashion. The numbers of mucus-containing cells were counted in more than 10 bronchioles in the 10 high-power fields per animal by measuring the length of epithelium defined along the basement membrane and luminal area using the NIH Image Analysis system [20]. Some lungs were stored at −80°C in paraffin blocks and stained with proteinase-activated receptor 2 (PAR-2) antibody (Santa Cruz Biotechnology, Santa Cruz, CA), and were examined under light microscopy (final magnification:x400). PAR-2 positive cells per square millimeter in the lung tissue were analyzed using NIH Image Analysis system. More than 10 bronchioles in a minimum of 10 high-power fields per lung tissue were randomly examined in a blinded fashion.

### Measurement of cytokine, chemokine and growth factor

The levels of cytokine, chemokine and growth factor in the BAL fluid and cell culture supernatants were measured by ELISA according to the manufacturer’s directions as previously described [21]. The limits of detection were 2 pg/ml for IL-4, KC and IFN-γ, 7 pg/ml for IL-5, 1.5 pg/ml for IL-13 and MIP-2, 3 pg/ml for eotaxin 4.61 pg/ml for TGF-β1 (R&D Systems, Minneapolis, MN).

### Measurement of serum anti-OVA antibody

Serum anti-OVA IgE antibody levels were measured by ELISA (DS pharma biomedical, Osaka, Japan), 6 and 48 hr after the last airway challenge as previously described [22]. The antibody titers of samples were related to pooled standards that were generated in the laboratory and expressed as ELISA (ng/ml). The limits of detection were 2.7 ng/ml.

### Flow cytometry

To stain intracellular protease-activated receptor 2 (PAR-2) in the peribronchial lymph nodes (PBLN), fixation and permeabilization of the cells were performed with BD cytofix/cytoperm kit (BD Biosciences Pharmingen, San Diego, CA, USA), and then incubated with anti-human PAR-2 mAb (Santa Cruz Biotechnology) according to the manufacturer’s directions. 1×10^6^ cells were incubated with PerCP-conjugated anti-CD3, PE-conjugated anti-human PAR-2 mAb or control antibody, FITC-conjugated anti-CD4 antibodies (BD Biosciences), and then 2×10^4^ cells were analyzed by flow cytometry (FACScalibur, Becton Dickinson Immunocytometry Systems) as previously described [23].

### Cell preparation and culture

Spleens from secondary challenged mice were removed and placed in PBS. Tissue was dispersed into single-cell suspensions, and mononuclear cells (MNC) were purified by Ficoll-Hypaque gradient centrifugation (Sigma-Aldrich) and cells (4×10^5^) were cultured for 24 hr in 96-well round-bottom plates in the presence or absence of OVA (100 μg/ml) as previously described [23].

### Statistical analysis

All results were expressed as mean ± SD. Multiple comparisons were performed by ANOVA following Newman-Keuls’s multiple comparison tests. The comparisons between two groups were performed by Mann–Whitney U-test. The p-value for significance was set at 0.05.

## Results

### Inhibition of neutrophil elastase attenuates AHR and airway inflammation 6 hr after secondary challenge

**Figure 1:**
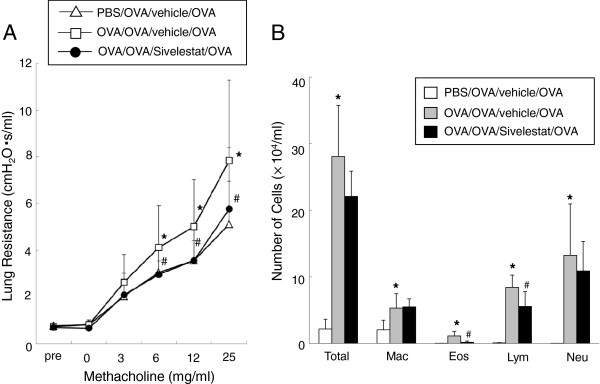
**Neutrophil elastase inhibitor attenuates AHR 6 hr after secondary challenge.** (**A**) Changes in R_L_ 6 hr after secondary challenge. R_L_ values to increasing concentrations of inhaled MCh were measured in non-sensitized/OVA-challenged mice receiving saline (PBS/OVA/vehicle/OVA), OVA-sensitized/OVA-challenged mice receiving saline (OVA/OVA/vehicle/OVA), and OVA-sensitized/OVA-challenged mice receiving sivelestat (OVA/OVA/Sivelestat/OVA). Results for each group are expressed as the mean ± SD. (n = 16-24 in each group). (**B**) Cell composition in BAL fluid obtained 6 hr after secondary challenge. Results for each group are expressed as the mean ± SD. (n = 16 in each group). *Significant differences (*P*<0.05) between PBS/OVA/OVA/vehicle and OVA/OVA/OVA/vehicle. #Significant differences (*P* < 0.05) between OVA/OVA/vehicle/OVA and OVA/OVA/Sivelestat/OVA.

Mice sensitized and challenged (primary) and mice challenged only were re-challenged (secondary) with OVA. Those mice which had previously been sensitized and challenged with OVA and treated with vehicle developed AHR compared to the non-sensitized but OVA challenged and re-challenged mice. When mice were treated with sivelestat, AHR were significantly reduced compared to vehicle saline-treated mice (Figure 1A). In parallel, inflammatory cell recruitment into the airways was increased 6 hrs after secondary airway challenge of previously sensitized and challenged animals (Figure 1B). Increased total cell numbers were largely due to increased numbers of neutrophils in BAL fluid (47% of total BAL fluid cells). When mice were treated with sivelestat, the numbers of eosinophils and lymphocytes were decreased significantly compared with vehicle-treated mice.

### Lung inflammation 6 hr after secondary challenge

**Figure 2:**
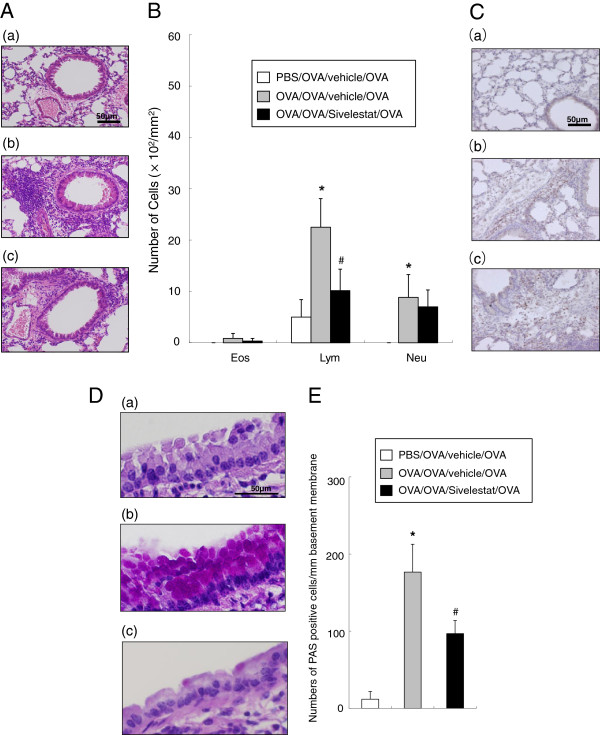
**Treatment with sivelestat reduces airway inflammation 6 hr after secondary challenge.** (**A**) H&E–stained lung tissue (final magnification:x400). (a) PBS/OVA/vehicle/OVA, (b) OVA/OVA/vehicle/OVA, (c) OVA/OVA/Sivelestat/OVA. (**B**) Inflammatory cell numbers in the peribronchial and perivascular tissue were measured (final magnification:x1000). (**C**) Anti-Gr-1 monoclonal antibody stained lung tissue (final magnification:x400). (a) PBS/OVA/vehicle/OVA, (b) OVA/OVA/vehicle/OVA, (c) OVA/OVA/Sivelestat/OVA. (**D**) Treatment with sivelestat suppresses goblet cell metaplasia. PAS staining was performed to identify mucus-containing cells in the airway epithelium (final magnification:x1000). (a) PBS/OVA/vehicle/OVA, (b) OVA/OVA/vehicle/OVA, (c) OVA/OVA/Sivelestat/OVA. (**E**) The number of mucus-positive cells. Data represent the mean ± SD. (n = 8 in each group). *Significant differences (*P*<0.05) between PBS/OVA/vehicle/OVA and OVA/OVA/vehicle/OVA. #Significant differences (*P*<0.05) between OVA/OVA/vehicle/OVA and OVA/OVA/Sivelestat/OVA.

In previous studies, the development of AHR was associated with inflammatory changes in lung tissue [24]. To determine if sivelestat affected inflammatory changes in the lung, we assessed tissue inflammation 6 hr after secondary OVA challenge. Hematoxylin-eosin and anti-Gr-1 monoclonal antibody stained lung tissue showed significant increases of neutrophil and lymphocyte numbers in peribronchial inflammation in previously sensitized and challenged animals compared to the non-sensitized mice. Mice treated with sivelestat demonstrated reduced the numbers of lymphocytes in lung tissue (Figure 2A, 2B, 2C).

Lung sections were stained with PAS to identify mucus-containing cells in the airway epithelium (Figure 2D). A significant increase in numbers of PAS positive cells was found in previously sensitized and challenged mice compared with non-sensitized but re-challenged mice. Treatment with sivelestat significantly reduced the number of PAS positive cells per millimeter of basement membrane (Figure 2E).

### Cytokines, chemokines and growth factor levels in BAL fluid 6 hr after secondary challenge

**Figure 3:**
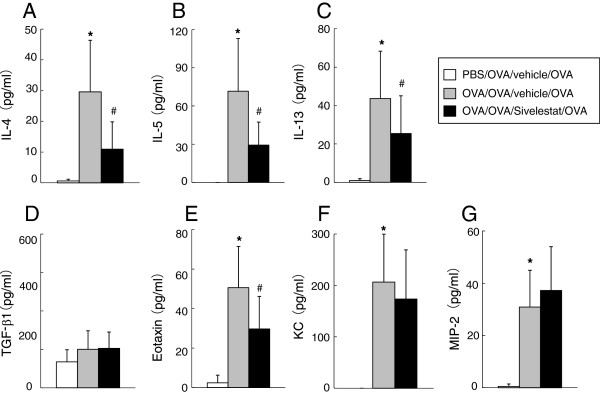
**Treatment with sivelestat alters cytokine, chemokine, and growth factor levels in BAL fluid 6 hr after secondary challenge.** The levels of (**A**) IL-4, (**B**) IL-5, (**C**) IL-13, (**D**) TGF-β1, (**E**) Eotaxin, (**F**) KC, and (**G**) MIP-2 in BAL fluid were measured. The results for each group are expressed as the mean ± SD. (n = 16 in each group). *Significant differences (*P* < 0.05) between PBS/OVA/vehicle/OVA and OVA/OVA/vehicle/OVA. #Significant differences (*P* < 0.05) between OVA/OVA/vehicle/OVA and OVA/OVA/Sivelestat/OVA.

Six hr after secondary allergen challenge, BAL fluid was obtained to assess cytokine and chemokine levels. After secondary challenge, Th2 (IL-4, IL-5 and IL-13) cytokines were all increased in sensitized and challenged mice treated with vehicle compared to non-sensitized mice. Treatment with sivelestat significantly reduced the levels of IL-4, IL-5 and IL-13 (Figure 3). Levels of eotaxin, KC, and MIP-2 in BAL fluid were also increased in sensitized and challenged mice treated with saline compared to non-sensitized mice, and treatment with Sivelestat significantly reduced the level of eotaxin but not KC or MIP-2 (Figure 3).

### Inhibition of neutrophil elastase prior to secondary challenge attenuates lung allergic responses 48 hr after secondary challenge

**Figure 4:**
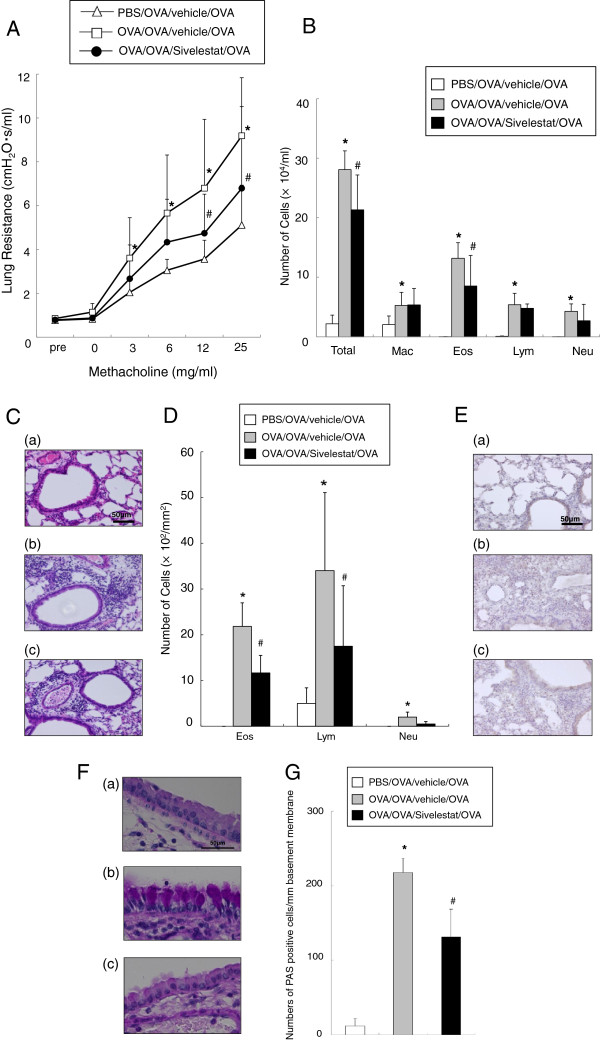
**Treatment with sivelestat reduces AHR and airway inflammation 48 hr after secondary challenge. (A**) Changes in R_L_ 48 hr after secondary challenge. Results for each group are expressed as the mean ± SD. **(**n = 16 in each group). (**B**) Cell composition in BAL fluid. Results for each group are expressed as the mean ± SD. (n = 16 in each group). (**C**) H&E–stained lung tissue (final magnification:x400). (a) PBS/OVA/vehicle/OVA, (b) OVA/OVA/vehicle/OVA, (c) OVA/OVA/Sivelestat/OVA. (**D**) Inflammatory cell numbers in the peribronchial and perivascular tissue (final magnification:x1000). (**E**) Anti-Gr-1 monoclonal antibody stained lung tissue (final magnification:x400). (a) PBS/OVA/vehicle/OVA, (b) OVA/OVA/vehicle/OVA, (c) OVA/OVA/Sivelestat/OVA. (**F**) PAS staining (final magnification:x1000). (a) PBS/OVA/vehicle/OVA, (b) OVA/OVA/vehicle/OVA, (c) OVA/OVA/Sivelestat/OVA. **(G**) The number of mucus-positive cells. Data represent the mean ± SD. (n = 8 in each group). *Significant differences (*P*<0.05) between PBS/OVA/vehicle/OVA and OVA/OVA/vehicle/OVA. #Significant differences (*P* < 0.05) between OVA/OVA/vehicle/OVA and OVA/OVA/Sivelestat/OVA.

We previously showed that at 48 hr after secondary allergen challenge, the inflammatory reaction and AHR developing after primary challenge resolved but that the re-challenge induced a strong inflammatory reaction with development of AHR [8]. Indeed, as observed with the increases in AHR 6 hr after the secondary challenge, these increases in AHR 48 hr after the secondary challenge were also significant (Figure 4A). Under these conditions, treatment with sivelestat significantly prevented the increases in AHR.

At 48 hr, increased total cell numbers were largely due to increased numbers of eosinophils and lymphocytes in the recovered BAL fluid. Administration of sivelestat at the time of the secondary challenge led to a significant decrease in eosinophil numbers in BAL fluid (Figure 4B). Sensitized and challenged mice treated with vehicle showed remarkable accumulation of the numbers of eosinophils and lymphocytes in lung tissue at 48 hr and administration of sivelestat significantly reduced the numbers of eosinophils and lymphocytes in lung tissue (Figure 4C, 4D, 4E) as well as numbers of goblet cells (Figure 4F, 4G).

**Figure 5:**
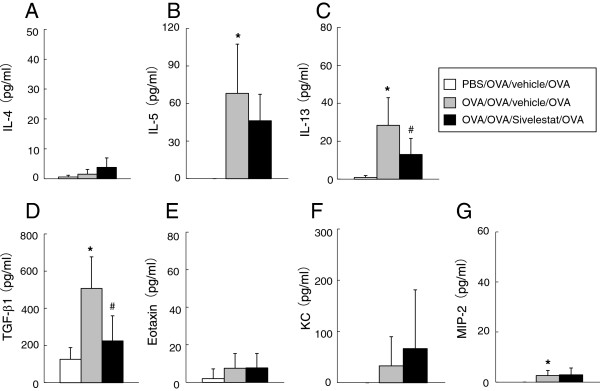
**Treatment with sivelestat alters cytokine and growth factor levels in BAL fluid 48 hr after secondary challenge.** The levels of (**A**) IL-4, (**B**) IL-5, (**C**) IL-13, (**D**) TGF-β1, (**E**) Eotaxin, (**F**) KC, and (**G**) MIP-2 in BAL fluid were measured. The results for each group are expressed as the mean ± SD. (n = 16 in each group). *Significant differences (*P*<0.05) between PBS/OVA/vehicle/OVA and OVA/OVA/vehicle/OVA. #Significant differences (*P* < 0.05) between OVA/OVA/vehicle/OVA and OVA/OVA/Sivelestat/OVA.

Forty-eight hr after the secondary challenge, IL-13 and IL-5 levels were increased in sensitized and challenged mice treated with vehicle compared to non-sensitized mice. Treatment with sivelestat significantly reduced IL-13 levels in BAL fluid. Levels of TGF-β1, and MIP-2 in BAL fluid were also increased in sensitized and challenged mice treated with vehicle compared to non-sensitized mice, and treatment with sivelestat significantly reduced the levels of TGF-β1 (Figure 5).

### Serum anti-OVA IgE antibody levels after secondary challenge

6 hr and 48 hr after secondary allergen challenge, serum was obtained to assess OVA-specific IgE levels. Levels of OVA-specific IgE were significantly increased in sensitized and challenged mice treated with vehicle compared with non-sensitized but challenged mice. Treatment with sivelestat did not affect serum OVA-specific IgE levels, likely since initial sensitization and challenge were completed before administration of the inhibitor (Table 1).

**Table 1:** Concentrations of OVA-specific IgE in the serum

	**6 hrs (ng/ml)**	**48 hrs (ng/ml)**
PBS/OVA/vehicle/OVA	5.5 ± 10.0	4.7 ± 9.4
OVA/OVA/vehicle/OVA	4682.1 ± 3919.8*	4182.8 ± 2819.6*
OVA/OVA/Sivelestat/OVA	4560.2 ± 2901.4	4855.6 ± 2101.6

Mice were sensitized and challenged as described in Methods. Serum levels of anti-OVA IgE antibody were assessed 6 h and 48 h after the last airway challenge. Mean values±SD are given (n = 8-12 in each group); PBS/OVA/vehicle/OVA: non-sensitized but challenged mice followed by treatment with vehicle; OVA/OVA/vehicle/OVA: sensitized and challenged mice followed by treatment with vehicle; OVA/OVA/Sivelestat/OVA: sensitized and challenged mice followed by treatment with sivelestat. *Significant differences (*P*<0.05) PBS/OVA/vehicle/OVA and OVA/OVA/vehicle/OVA.

### PAR-2 expression in lung tissue

**Figure 6:**
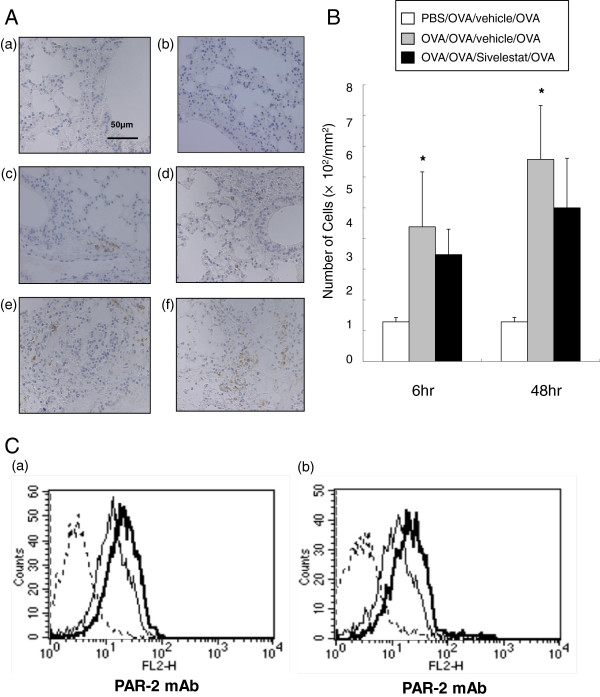
**PAR-2 expression on lung tissue and T cells in PBLN.** (**A**) PAR-2 stained lung tissue obtained 6hr and 48 hr after secondary challenge (final magnification:x400). (a) 6 hr: PBS/OVA/vehicle/OVA, (b) 48 hr:PBS/OVA/vehicle/OVA, (c) 6 hr: OVA/OVA/vehicle/OVA, (d) 6 hr:OVA/OVA/Sivelestat/OVA, (e) 48 hr: OVA/OVA/vehicle/OVA, (f) 48 hr: OVA/OVA/Sivelestat/OVA. (**B**) The number of PAR-2-positive cells. *Significant differences (*P*<0.05) between PBS/OVA/vehicle/OVA and OVA/OVA/vehicle/OVA. Data represent the mean ± SD (n = 6 in each group). (**C**) Dotted line: Control antibody, Thin line: anti-human PAR-2 mAb, Non/Non/Non, Bold line: anti-human PAR-2 mAb, OVA/OVA/OVA. PAR-2 expression of CD3^+^ and CD4^+^ T Cells in PBLN. (a) CD3^+^ T cells in the PBLN and (b) CD4^+^ T Cells in PBLN were assessed by intracellular staining. The data shown are representative of three independent experiments. Increased numbers of PAR-2 positive CD3^+^ and CD4^+^ T cells from the PBLN of the sensitized and challenged mice were observed.

PAR-2 has been reported to be one of the receptors for neutrophil elastase [25], and is expressed on a variety of cells including airway epithelial cells, fibroblasts, myocytes, sensory neurons, and bronchial and vascular smooth muscle [26,27]. PAR-2 was detected intracellularly in eosinophils but at undetectable levels on the cell surface. However, once these few receptors became activated, PAR-2 was redistributed from intracellular stores to the surface of the cell [28]. In lung tissue assessed for PAR-2 staining, non-sensitized and challenged mice showed few PAR-2 positive cells (Figure 6A(a), 6A(b), 6B), whereas sensitized and challenged and both 6 and 48 hr after secondary allergen challenged mice showed increased numbers of PAR-2 positive cells (Figure 6A(c), 6A(e), 6B). Treatment with sivelestat did not affect the number of PAR-2 positive cells (Figure 6A(d), 6A(f), 6B).

### PAR-2 expression on PBLN T cells

To determine the expression of PAR-2 in T cells, percentages of PAR-2-positive CD3^+^ and CD4^+^ T cells in PBLN were assessed by intracellular staining. As shown in Figure 6C, increased numbers of PAR-2 positive CD3^+^ and CD4^+^ T cells were observed in the PBLN of sensitized and challenged and secondary challenged mice. Sivelestat did not have any significant effect on the number of PAR-2 positive CD3^+^ and CD4^+^ T cells in the PBLN (data not shown).

### Effect of neutrophil elastase inhibition on in vitro cytokine production from spleen cells

**Figure 7:**
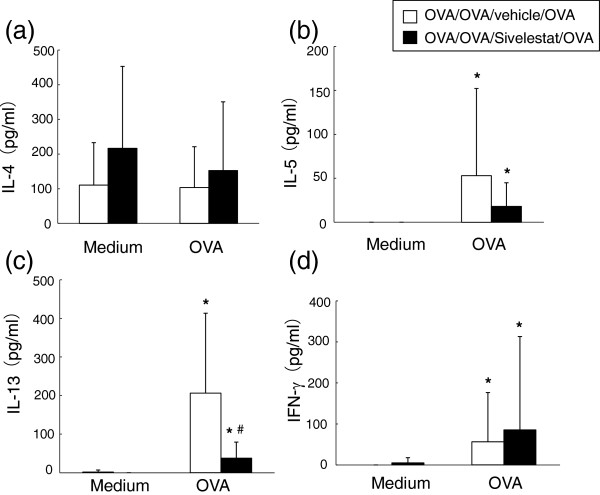
**Effect of neutrophil elastase inhibitor for in vitro cytokines production.** In vitro cytokine production from spleen cells obtained 6 h after secondary challenge. The levels of (**a**) IL-4, (**b**) IL-5, (**c**) IL-13 and (**d**) IFN-γ in culture supernatant of spleen cells from mice after OVA sensitization and challenge were measured. The results for each group are expressed as the mean ± SD. (n = 12 in each group). *P < 0.05 without re-stimulation with OVA groups (medium) vs. with re-stimulation with OVA groups. #Significant differences (*P* < 0.05) between OVA/OVA/vehicle/OVA and OVA/OVA/Sivelestat/OVA.

To determine whether the difference in cytokine levels observed in the BAL fluid of mice treated with sivelestat were due to a difference in antigen-specific T-cell responsiveness, spleen cells were isolated 6 hr after secondary OVA challenge, and re-stimulated in culture for 24 hrs with OVA. There were no significant differences in cultures of cells from mice treated with sivelestat and those treated with saline in for IFN-γ production. After culture with OVA, spleen cells from mice treated with sivelestat secreted significantly lower amounts of IL-13 than did spleen cells from mice treated with vehicle (Figure 7).

## Discussion

The main finding of this study is that a specific inhibitor of neutrophil elastase, sivelestat, influences the lymphocytes, which produce less IL-13, resulting in a decreased AHR and airway inflammation. In the present study, we evaluated the role of neutrophil elastase in allergen-induced inflammation and AHR on a background of previously established disease, provoked by secondary challenge. This model perhaps more closely mimics the human situation of previous exposure than primary models of acute exposure. We demonstrated that treatment with sivelestat administered after primary sensitization and challenge but prior to secondary challenge effectively prevented the triggering of AHR, eosinophilic inflammation, Th2 cytokine production and goblet cell metaplasia. The effects were similar whether examined at 6 hr during a stage characterized by neutrophilic inflammation and at 48 hr when eosinophilic inflammation predominated.

Administration of house dust mite (HDM) by inhalation was shown to induce airway inflammation without systemic immunization. Rydell-Tormanen et al. demonstrated that 20 weeks of HDM extract exposure resulted in a reduction in the proportion of eosinophils and an increase in neutrophils compared with the inflammatory response induced by 7 weeks, and that HDM extract exposure induced airway and vascular remodeling [29]. Unlike OVA, HDM extract is complex materials consisting of many protein and non-protein components, which are biochemically active and may play a role in enhancing Th2 immune responses. HDM allergens have proteinase activity, which is critical for sensitization, and react with toll-like receptor 4 (TLR4) [30,31]. In addition, the group 2 major mite allergen (Der f2) possesses structural homology to myeloid differentiation factor (MD) 2, the lipopolysaccharide (LPS)-binding component of the TLR4 signaling complex [32,33]. These findings indicate that HDM allergen sensitization still involves complex interactions between antigen-specific responses and innate immune responses that have not yet been clarified. On the other hand, recent studies demonstrated that different OVA-mouse models induced the neutrophilic allergic airway inflammation. Bobic et al. have shown that IL-13 and IL-17 levels and the total cell counts in BAL fluid were increased with higher neutrophil as well as eosinophil, lymphocyte in Balb/c mice which were sensitized with OVA by seven intraperitoneal injections and exposed to aerosolized OVA for 8 subsequent days [34]. Nabe et al. have reported that in Balb/c mice which were sensitized with OVA on days 0, 14 and 28, and challenged by intratracheal administration of OVA on days 35, 36, 37 and 40, the numbers of neutrophils, which increased before and after the 2nd and 3rd challenges, returned towards baseline prior to the 4th challenge, but showed recurrent airway neutrophilia after the 4th challenge. Furthermore, systemic treatment with the anti-Gr-1 monoclonal antibody markedly suppressed 4th challenge-induced airway neutrophilia and the induction of a late-phase increase in AHR [35]. In the present study, mice developed a two-phase airway inflammatory response after secondary allergen challenge, one neutrophilic at 6 hr and the other eosinophilic, at 48 hr. AHR to inhaled MCh was detected at both phases of the response to secondary challenge. In the first phase, 6 hr after last antigen challenge, mice developed AHR and a neutrophil-dominant airway inflammatory response with relatively small numbers of lymphocytes and eosinophils in the BAL fluid. Administration of the neutrophil elastase inhibitor, sivelestat, reduced AHR and the number of eosinophils and lymphocytes in the airways. Of note, the numbers of neutrophils in BAL fluid were only marginally reduced. In asthmatics [36] and in animal models [37], neutrophils have been shown to be the major inflammatory cells in the airways early after allergen challenge. The timing of the peak neutrophil influx coincided with development of AHR. The response to antigen challenge at 48 hrs was characterized by a marked increase in numbers of eosinophils, also accompanied by development of AHR. Treatment with sivelestat similarly reduced the numbers of eosinophils and suppressed AHR at this point in time. Thus, inhibition of neutrophil elastase may represent a novel therapeutic target. The results of our study differ somewhat from with a previous study showing that neutrophil elastase contributes to asthmatic responses where a different sheep model of allergen-induced airway responses was assessed using, nonetheless neutrophil elastase inhibitor but did not affect the number of eosinophils and lymphocytes in BAL fluid [18]. The basis for this discrepancy is not clear but may reflect the use of a totally different protocol as well as model, using Ascaris suum antigen in sheep. Moreover, the levels of cytokine, chemokine and growth factors in the BAL fluid were not measured in the study.

To address the underlying mechanisms whereby neutrophil elastase inhibition affects allergen-induced airway inflammation and AHR, BAL cytokine levels were assayed. Although sivelestat did not affect levels of the neutrophil chemoattractant, KC or MIP-2, it significantly reduced the levels of BAL Th2 type cytokines, IL-4, IL-5 and IL-13, and eotaxin in BAL fluid 6 hr after secondary allergen challenge. At 48 hr, treatment with sivelestat significantly reduced the levels of IL-13 and TGF-β1 in the BAL fluid. Assessment of in vitro Th2 cytokine production from spleen cells after re-stimulation with OVA confirmed that cells obtained from the mice which received sivelestat treatment also produced lower levels of IL-13 whereas levels of IFN-γ were unaffected. These data suggest that inhibition of neutrophil elastase affects Th2 cytokine production which, in turn, leads to reduction in allergic airway responses.

Protease-activated receptors (PARs) are a novel family of G-protein-coupled receptors that are activated upon cleavage of the N terminus of the receptor by proteases. This cleavage exposes a previously cryptic, tethered ligand, which then binds intramolecularly to the second extracellular loop to activate the associated G-protein [38,39]. PARs are expressed on a variety of cells including platelets, eosinophils, neutrophils, mononuclear cells and epithelial cells in the airway [40]. PAR-2 is one of the receptors for the neutrophil elastase, which has been reported to mediate eosinophil infiltration and AHR [41]. Neutrophil serine proteinases activate human nonepithelial cells to produce inflammatory cytokines through PAR-2 [25]. Since eosinophils express PAR-2 intracellularly [28], neutrophil-derived serine proteases may activate eosinophils [42]. We found that PAR-2 expression in the lung tissues was enhanced following secondary challenge, and that PAR-2 intracellular expression on PBLN T cells was also increased following allergen challenge of sensitized mice, suggesting the involvement of PAR-2 in allergic airway responses. In the present study, treatment with a specific inhibitor of neutrophil elastase, sivelestat, did not alter the number of PAR-2 positive cells, nonetheless a PAR-2-neutrophil elastase pathway may play an important role in allergic inflammation since sivelestat effectively prevented the triggering of AHR, eosinophilic inflammation, Th2 cytokine production. Therefore, additional mechanisms beyond PAR-2 positive cell numbers but which involve PAR-2 pathways critical to the development of allergic airway responses need further investigation.

Interestingly, Kikuchi et al. have shown that neutrophils enhance the trans-basement membrane migration of eosinophils in vitro, therefore, activation of neutrophils may enhance the accumulation of eosinophils in the airways, sustaining allergic inflammation [43]. LTB4, a lipid mediator that is derived from membrane phospholipid, is thought to play an important role in the activation and recruitment of leukocytes, including neutrophils [44]. We have recently shown that chemoattraction and activation of neutrophils through LTB4-BLT1 may contribute, at least in part, to allergic airway inflammation in established asthma [45]. Further understanding of the relationship between neutrophils and eosinophils, and between LTB4-BLT1 and the PAR-2-neutrophil elastase pathway may help clarify the complicated mechanisms of asthma development.

Mouse eotaxin has been shown to be a potent chemoattractant for eosinophils during inflammation and allergic reactions [46]. Eotaxin production by bronchial epithelial cells was up-regulated by IL-4 and IL-13, and attenuated by IFN-γ. In this study, eotaxin levels in BAL fluid were increased 6 hrs after allergen re-exposure in previously sensitized mice and significantly decreased by treatment with the neutrophil elastase inhibitor. Inhibition of IL-4 and IL-13 production by the neutrophil elastase inhibitor may down-regulate eotaxin secretion, thus suppressing migration of eosinophils to the airways.

After treatment with the neutrophil elastase inhibitor, the levels of TGF-β1 in BAL fluid were also decreased. It has been shown that IL-13 induces tissue fibrosis by selectively stimulating and activating TGF-β1 and that IL-13 action in concert with TGF-β1 may increase the release of eotaxin from human fibroblasts [47,48]. Minshall et al. and ourselves demonstrated that TGF-β1 might play a role in the fibrotic changes occurring within asthmatic airways, and that activated eosinophils were a major source of this cytokine [49,50]. Thus, manipulating neutrophil elastase may be effective for reducing airway fibrotic changes and remodeling through suppression of eosinophil activation and TGF-β1 secretion.

## Conclusion

Administration of the neutrophil elastase inhibitor, sivelestat, reduced AHR slightly but significantly in mice with established airway disease and to reduce BAL fluid Th2 cytokine, eotaxin and TGF-β1 levels and goblet cell metaplasia. This was accompanied by reducing eosinophils in the airways at the two distinct phases of the response to secondary allergen challenge, but not specific IgE level. Taken together, in both the neutrophil- and eosinophil-dominant phases of the response to secondary allergen challenge, the neutrophil elastase inhibitor reduced AHR to inhaled methacholine indicating the potential for its use as a modulator of the immune/inflammatory response in established asthma.

## Abbreviations

AHR: Airway hyperresponsiveness; BAL: Bronchoalveolar lavage; MCh: Methacholine; OVA: Ovalbumin; PBLN: Peribronchial lymph node; RL: Lung resistance.

## Competing interests

The authors declare that they have no competing interests.

## Authors’ contributions

Study conception and design: HK, AK. Performed experiments, data analysis: HK, YF, GI, KW, KO, YT, MK, MT. Manuscript writing and editing: HK, NM, EG, AK. All authors read and approved the final manuscript.
